# Glutamine metabolism in adipocytes: a *bona fide* epigenetic modulator of inflammation

**DOI:** 10.1080/21623945.2020.1831825

**Published:** 2020-10-12

**Authors:** Simon Lecoutre, Salwan Maqdasy, Paul Petrus, Alison Ludzki, Morgane Couchet, Niklas Mejhert, Mikael Rydén

**Affiliations:** a Department of Medicine (H7), Karolinska University Hospital, Stockholm, Sweden; b CHU Clermont-Ferrand, Service D’endocrinologie, Diabétologie et Maladies Métaboliques, Clermont-Ferrand, France; c Faculté de Médecine, Laboratoire GReD, Université Clermont Auvergne, Clermont-Ferrand, France; d Center for Epigenetics and Metabolism, Department of Biological Chemistry, INSERM U1233, University of California, Irvine, CA, USA

**Keywords:** metabolite, adipocyte, inflammation, insulin resistance, epigenetics, amino acids

## Abstract

A chronic low-grade inflammation of white adipose tissue (WAT) is one of the hallmarks of obesity and is proposed to contribute to insulin resistance and type 2 diabetes. Despite this, the causal mechanisms underlying WAT inflammation remain unclear. Based on metabolomic analyses of human WAT, Petrus et al. showed that the amino acid glutamine was the most markedly reduced polar metabolite in the obese state. Reduced glutamine levels in adipocytes induce an increase of Uridine diphosphate N-acetylglucosamine (UDP-GlcNAc) levels via induction of glycolysis and the hexosamine biosynthetic pathways. This promotes nuclear O-GlcNAcylation, a posttranslational modification that activates the transcription of pro-inflammatory genes. Conversely, glutamine supplementation in vitro and in vivo, reversed these effects. Altogether, dysregulation of intracellular glutamine metabolism in WAT establishes an epigenetic link between adipocytes and inflammation. This commentary discusses these findings and their possibly therapeutic relevance in relation to insulin resistance and type 2 diabetes.

## Abstract

A chronic low-grade inflammation of white adipose tissue (WAT) is one of the hallmarks of obesity and is proposed to contribute to insulin resistance and type 2 diabetes. Despite this, the causal mechanisms underlying WAT inflammation remain unclear. Based on metabolomic analyses of human WAT, Petrus et al. showed that the amino acid glutamine was the most markedly reduced polar metabolite in the obese state. Reduced glutamine levels in adipocytes induce an increase of Uridine diphosphate N-acetylglucosamine (UDP-GlcNAc) levels *via* induction of glycolysis and the hexosamine biosynthetic pathways. This promotes nuclear O-GlcNAcylation, a posttranslational modification that activates the transcription of pro-inflammatory genes. Conversely, glutamine supplementation *in vitro* and *in vivo*, reversed these effects. Altogether, dysregulation of intracellular glutamine metabolism in WAT establishes an epigenetic link between adipocytes and inflammation. This commentary discusses these findings and their possibly therapeutic relevance in relation to insulin resistance and type 2 diabetes. (Word count:148)

## Introduction

White adipose tissue (WAT) is a highly dynamic organ that can rapidly expand in response to caloric oversupply. When energy intake exceeds energetic requirements, adipocytes take up nutrients from the circulation and store them as triglycerides within a unilocular cytoplasmic lipid droplet. In addition to storing lipids, adipocytes are energy sensors that secrete adipocytokines as well as metabolites which together impact on multiple physiological processes such as hepatic gluconeogenesis, cell proliferation/tissue growth, blood pressure, angiogenesis, coagulation, chemotaxis, and inflammation (reviewed in [1]). Much research has focused on the chronic low-grade inflammation of WAT associated with obesity and insulin resistance. Thus, in the hypercaloric state adipocytes secrete TNFα, IL-6 and other pro-inflammatory cytokines that are important for healthy WAT remodelling and expansion [2]. Hence, the initial phase of WAT expansion is characterized by a transient phase of inflammation which in turn is considered to be an adaptive response with beneficial effects on tissue dynamics [2,3]. However, a constant caloric oversupply and expansion of WAT is associated with a non-resolving immune activation which over time becomes maladaptive. This chronic pro-inflammatory state is capable of resetting homoeostatic set-points thereby prompting the development of insulin resistance, in part *via* ectopic lipid deposition in liver and muscles [4,5]. Yet, our understanding of the events that initiate WAT inflammation in obesity is limited, and the causal relationship between inflammation and the complications of obesity remains a subject of controversy.

## Chromatin remodelling links metabolism to inflammation

It has become increasingly evident in the field of immunology that metabolism and energetic states of immune cells regulate inflammation *via* epigenetic modifications (reviewed in [6]). Thus, metabolites from multiple pathways can remodel the chromatin via different mechanisms and adapt the transcriptional programs to a specific metabolic state. This interaction is therefore crucial to coordinate cellular function and determine cellular identity. Mechanistically, many metabolites constitute substrates or co-substrates for enzymes that regulate gene expression through DNA methylation and post-translational modifications of histones. For instance, acetyl-CoA, produced mainly through glycolysis, β-oxidation and glutaminolysis, serves as an ‘acetyl’ group donor for protein acetyltransferase, a critical step during protein acetylation [6]. Nicotinamide adenine dinucleotide (NAD+) is an essential cofactor for some histone deacetylases (sirtuins) [7]. Alpha‑ketoglutarate (αKG), a metabolite of the TCA cycle and glutamine metabolism, is a crucial co-factor for DNA and histone demethylation by JMJD histone demethylases and 5‑methylcytosine hydroxylases (TETs) [8,9]. Other specific metabolites involved in chromatin remodelling include malonyl-CoA for histone malonylation, lactate for histone lactylation and UDP-GlcNAc for histone GlcNAcylation [10,11,12].

The connections between cellular metabolism and epigenetic modifications lead to altered gene transcription. One example of this occurs in pro-inflammatory M1 macrophages which are metabolically characterized by increased glucose uptake and enhanced aerobic glycolysis (reviewed in [13]). In this setting, pyruvate produced by the glycolytic pathway, can either be funnelled into lactate production or transported into mitochondria where it is converted to acetyl-CoA which then feeds into the TCA cycle. Increased TCA cycle activity leads to the accumulation of metabolites like citrate, itaconate and succinate and an increase in ROS production [14]. Lauterbach and colleagues showed that the production of citrate after TLR4 activation results in increased levels of acetyl-CoA *via* the activity of ATP citrate lyase which promotes histone acetylation. The later triggers the expression of genes encoding pro-inflammatory cytokines [15]. Moreover, increased intracellular lactate levels can also induce histone modification (lysine lactylation) 16–24 h after exposure to M1-polarizing stimuli [11]. Histone lactylation is not required for the pro-inflammatory gene expression; instead, it is implicated in the upregulation of homoeostatic genes that counteract the inflammatory reaction [11].

## A link between glutamine metabolism and chromatin remodelling

Recent studies have shown that the interplay between glycolysis and glutamine metabolism is crucial for the induction of the epigenetic and functional changes in immune cells through fumarate accumulation. The latter is driven by enhanced feeding of glutamine into the TCA cycle through glutaminolysis [16]. Thus, Arts et al. demonstrated that the accumulation of fumarate in β-glucan-treated monocytes was essential for trained immunity [16]. Fumarate accumulation induced epigenetic changes by inhibition of H3K4 demethylase KDM5A, thereby increasing H3K4me3 histone modification (i.e. permissive chromatin) and promoting pro-inflammatory cytokine production upon re-stimulation with LPS [16]. These findings are in line with the role of glutamine metabolism in immune cell differentiation [17,18]. Indeed, glutaminolysis induced by IL4, leads to increased αKG levels, a substrate for the histone demethylase JMJD3. This enzyme allows demethylation of H3K27 and hence, full activation M2-associated genes [17]. Moreover, in T cells, glutaminase activity suppresses effector T cell differentiation and function [18]. Altogether, this demonstrates that glutamine metabolism plays a key role in the regulation of the inflammatory response of immune cells.

## The immunomodulatory potential of glutamine in adipose tissue

In contrast to immune cells, very little is known about the interplay between metabolism and chromatin remodelling in adipocytes. Recently, *Petrus* et al. reported that altered glutamine metabolism in hypertrophic adipocytes, modulates the expression of proinflammatory pathways in white adipocytes [19]. In an untargeted analysis of polar metabolites from human WAT, glutamine was one of the most significantly altered metabolites in obesity. Analyses in different clinical cohorts revealed that the reduction of glutamine was closely linked to different parameters related to WAT inflammation. A causal relationship was suggested by the observation that intra-peritoneal administration of glutamine in high fat diet-fed mice decreased WAT inflammation and macrophage infiltration. Attenuated WAT glutamine levels in obesity were linked to reduced glutamine synthetase (*GLUL*) expression in several cell types although the reduction was most prominent in adipocytes [19]. Anti-inflammatory effects of GLUL-mediated glutamine production have been described in several cell types including murine microglia [20], T cells [18,21,22] and macrophages [23,24], as well as adipocytes differentiated from a murine cell line [23].

Given that metabolic pathways are interconnected, and metabolite fluxes change over time, it was hypothesized that dysregulation of glutamine metabolism involved a global metabolic reprogramming of WAT. By combining metabolomics and bioenergetic analyses of human *in vitro* differentiated adipocytes, Petrus et al. observed that glutamine inhibits glycolysis and the hexosamine biosynthetic pathway resulting in a reduction of UDP-GlcNAc levels. This metabolite is the only substrate for the enzyme O-GlcNAc transferase enzyme (OGT), which catalyzes the post-translational modification of protein targets by adding O-GlcNAc to serine or threonine residues. O-GlcNAcase (OGA) catalyzes the hydrolysis of this sugar modification [25]. Unlike traditional glycosylation, protein O-GlcNAcylation can occur in the cytoplasm, nucleus, and mitochondria and it is an essential post-translational modification allowing dynamic control of protein stability, localization, transcriptional activity and multiple other cellular functions [26]. Numerous studies have shown that transcription factors and cofactors are modified by O-GlcNAcylation [27]. The pathophysiological relevance of this epigenetic modification is supported by several studies reporting that perturbations in cellular O-GlcNAc homoeostasis are linked to a plethora of human diseases, including the pathogenesis of both type-1 and type-2 diabetes [28].

**Figure 1.**
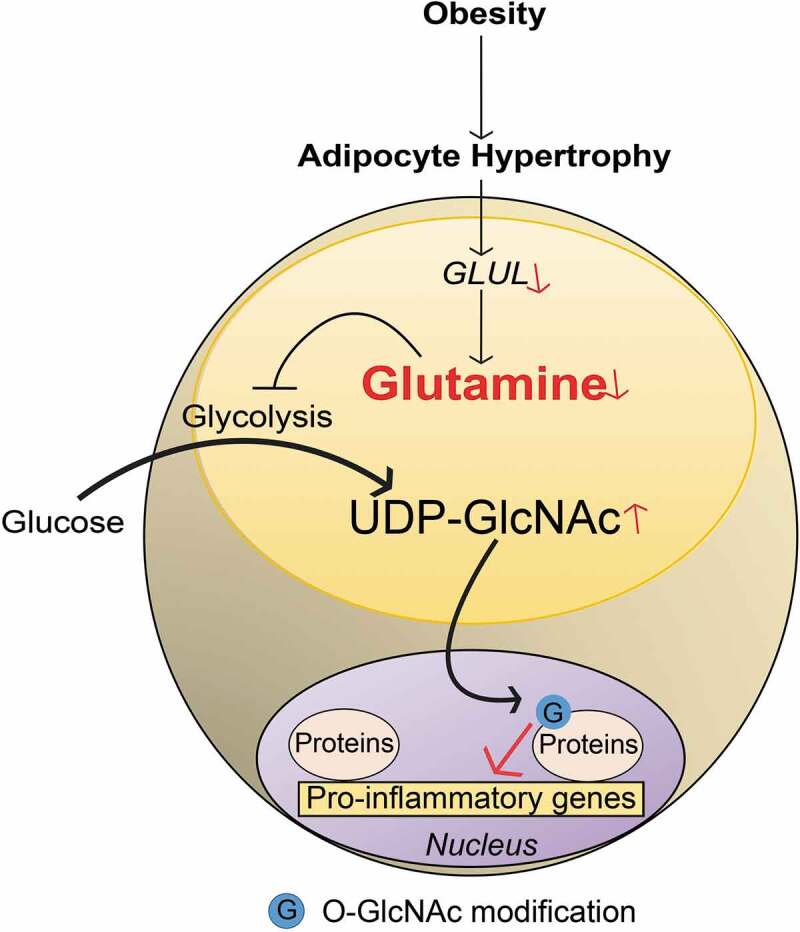
Linking glutamine metabolism to inflammation in obesity. A schematic representation summarizing the findings discussed in this commentary. In obesity, adipocyte hypertrophy attenuates *GLUL* expression via unclear mechanisms. This reduces the conversion of glutamate to glutamine resulting in reduced intracellular glutamine levels. Lowered glutamine levels shift the balance from glutaminolysis towards glycolysis, leading to increased activity in hexosamine biosynthetic pathway and higher levels of UDP-GlcNAc. The latter promotes nuclear O-GlcNAcylation (symbolized by G, e.g. SP1) which increases the transcriptional activity of pro-inflammatory genes

The possible mechanisms through which glutamine and O-GlcNAcylation might regulate gene expression was explored in human *in vitro* differentiated adipocytes. This demonstrated that glutamine reduced UDP-GlcNAc levels and decreased nuclear protein O-GlcNAcylation in the promoter regions of pro-inflammatory genes. This effect was counteracted by treatment with an OGA inhibitor. A genome-wide *de novo* motif analysis of the chromatin regions displaying glutamine-linked changes in O-GlcNAcylation revealed an enrichment of the binding motif for SP1, a transcription factor that plays a central role in the regulation of pro-inflammatory responses. It has been shown in other cell systems that O-GlcNAcylation modulates SP1 activity and plays a role in hyperglycaemia-induced inflammation [29]. By immunoprecipitation of O-GlcNAc-modified proteins isolated from nuclei of human adipocytes, Petrus et al. confirmed that glutamine reduced both SP1 O-GlcNAcylation and the expression of SP1-regulated target genes. Importantly, SP1 O-GlcNAcylation modified by glutamine is only one example and a wide range of additional O-GlcNAcylated proteins may be regulated by glutamine. Nevertheless, this establishes a link between glutamine, O-GlcNAcylation and inflammatory response and provides the basis for future studies to understand the regulation of nuclear O-GlcNAcylation and its broader role in gene regulation (Figure 1).

## The unresolved link between glutamine and glycolysis

The findings above demonstrate that glutamine inhibits glycolysis and thereby the hexosamine pathway. An interaction between glycolysis and glutamine metabolism has been known for long but how these two pathways influence each other remains unclear [30]. Previous studies have suggested that the crosstalk is partially mediated by metabolites such as NAD(P)H, alpha ketoglutarate, succinate and/or citrate (reviewed in [31]). These metabolites can act as cofactors, controlling directly (allosterically) or indirectly (*via* AMPK, mTORC, HIF1) the activity/expression of specific enzymes involved in glucose and glutamine metabolism (reviewed in [31]). Nevertheless, future studies are required to establish the direct link between glutamine and glycolysis in white adipocytes.

## Clinical implications and perspectives

Altogether, these data demonstrate that, similar to immune cells, adipocytes sense changes in the metabolic state resulting in an inflammatory response. Given the strong correlation between WAT glutamine levels (and *GLUL* expression) and adipocyte volume, it is conceivable that reduced local glutamine levels secondary to adipocyte hypertrophy activate inflammation to promote WAT expansion. This response is dynamic; it is reversed once energy homoeostasis has been achieved but becomes maladaptive in conditions of constant caloric oversupply. The potential clinical implication of glutamine has been studied in a plethora of conditions ranging from critical illnesses [32] to the metabolic syndrome [33] and type 2 diabetes [34]. Short-term glutamine supplementation is associated with reductions in body weight and fat mass as well as improved insulin sensitivity and glucose homoeostasis [33,35]. Moreover, it results in higher incretin levels promoting insulin secretion in type 2 diabetic patients [36]. Glutamine may therefore constitute an interesting therapeutic compound to reduce WAT inflammation, a cornerstone in type 2 diabetes pathophysiology. In summary, the results by Petrus et al, highlight novel aspects on the molecular mechanisms driving inflammation suggesting that glutamine plays a key role in regulating obesity-associated inflammation in WAT. In this regard, it will be important to identify the transcriptional regulatory network that controls glutamine metabolism in WAT and why it is perturbed in the obese state. Future investigations are needed to understand the dynamics of this immunometabolic crosstalk. Ultimately, these insights might offer new therapeutic perspectives and identify nutritional interventions that target WAT inflammation and insulin resistance.
